# 771 Improving Burn Care in the Amish Community: A Qualitative Study of Strengths and Needs

**DOI:** 10.1093/jbcr/irac012.324

**Published:** 2022-03-23

**Authors:** Jamey T Brumbaugh, Jennifer A Kelleher, Mira Snider, Mariah Sturges, Ariel M Aballay, Christina L Duncan

**Affiliations:** West Virginia University, Pittsburgh, Pennsylvania; West Virginia University, Morgantown, West Virginia; Ann & Robert H. Lurie Children's Hospital of Chicago, Chicago, Illinois; AHN West Penn, Pittsburgh, Pennsylvania; West Penn Hospital, Allison Park, Pennsylvania; West Virginia University, Morgantown, West Virginia

## Abstract

**Introduction:**

Amish communities present with a higher risk for sustaining traumatic burn injuries; thus, these communities have a high need for culturally competent burn care. Although homeopathic remedies for mild to moderate burn injuries have been studied in Amish communities, little is known about hospital-community partnerships to facilitate culturally competent burn care, particularly with more severe injuries. The current study aimed to 1) understand the successful aspects of an existing hospital-community partnership for facilitating culturally sensitive burn care for Amish communities, and (2) identify the ongoing physical, structural, and behavioral health needs of this population as the partnership continues to develop.

**Methods:**

Qualitative data from 12 Amish caregivers who participate on a burn/wound team or an oil therapy team, were collected on through a focus group interview. Caregivers identified as White, were majority male (83%), and resided in Amish communities. Retrospective thematic analysis was used to analyze the qualitative data. Five major themes (i.e., informational needs, strengths of Amish burn care, behavioral health concerns, behavioral health resources, and preferred teaching methods) evolved.

**Results:**

Results indicated that Amish caregivers displayed a great curiosity and openness to learning about all aspects of recommended burn care from the medical providers. Caregivers also cited their traditional (homeopathic) burn care procedures (e.g., oil therapy) as strengths, while simultaneously maintaining that their relationship to the hospital is a valuable part of their burn care. Relating to behavioral health, caregivers highlighted difficulties in helping their children cope with burn injuries and pain during rehabilitation and treatment. Caregivers emphasized the role of strong social support that the Amish community provides to burn survivors. Additionally, caregivers stressed the significance of delivering burn care information in a form that is culturally appropriate for their community.

**Conclusions:**

Results of this study provide important considerations that other accredited burn centers may consider when establishing similar partnerships to enhance their delivery of culturally competent medicine for Amish burn survivors.

